# Insulator–metal transition in substrate-independent VO_2_ thin film for phase-change devices

**DOI:** 10.1038/s41598-017-17937-3

**Published:** 2017-12-20

**Authors:** Mohammad Taha, Sumeet Walia, Taimur Ahmed, Daniel Headland, Withawat Withayachumnankul, Sharath Sriram, Madhu Bhaskaran

**Affiliations:** 1 Functional Materials and Microsystems Research Group and Micro Nano Research Facility, RMIT University, Melbourne, Victoria, 3001 Australia; 2 School of Electrical and Electronic Engineering, The University of Adelaide, South Australia, 5005 Australia

**Keywords:** Electronic properties and materials, Electronic devices

## Abstract

Vanadium has 11 oxide phases, with the binary VO_2_ presenting stimuli-dependent phase transitions that manifest as switchable electronic and optical features. An elevated temperature induces an insulator–to–metal transition (IMT) as the crystal reorients from a monoclinic state (insulator) to a tetragonal arrangement (metallic). This transition is accompanied by a simultaneous change in optical properties making VO_2_ a versatile optoelectronic material. However, its deployment in scalable devices suffers because of the requirement of specialised substrates to retain the functionality of the material. Sensitivity to oxygen concentration and larger-scale VO_2_ synthesis have also been standing issues in VO_2_ fabrication. Here, we address these major challenges in harnessing the functionality in VO_2_ by demonstrating an approach that enables crystalline, switchable VO_2_ on any substrate. Glass, silicon, and quartz are used as model platforms to show the effectiveness of the process. Temperature-dependent electrical and optical characterisation is used demonstrating three to four orders of magnitude in resistive switching, >60% chromic discrimination at infrared wavelengths, and terahertz property extraction. This capability will significantly broaden the horizon of applications that have been envisioned but remained unrealised due to the lack of ability to realise VO_2_ on any substrate, thereby exploiting its untapped potential.

## Introduction

Vanadium dioxide (VO_2_) presents a characteristic temperature-dependent insulator–to–metal transition (IMT). While other materials (including other oxides of vanadium) demonstrate IMT, VO_2_ is significant for switching near an electronics-compatible temperature of ~68 °C^1–6^. This change in electrical properties is accompanied by a simultaneous transition in optical properties, wherein the material changes from being transparent to nearly opaque at infrared (IR) wavelengths. As a result, the temperature-dependent transition in electrical and optical properties of VO_2_ can be exploited for a range of applications such as smart windows^7–9^, electro-optic modulators^10^, memory devices^11^, terahertz systems^12,13^, thermal actuators^14^, Mott transistors^15^, strain sensors^16^, and thermo/electrochromic layers^17^. Significantly, the ability to synthesis high quality VO_2_ films has greater consequences as doping with W or Mo can lower the IMT temperature closer to ambient conditions^18–20^.

While the relationship between the stoichiometry, microstructure, and the nature and degree of the IMT is still a topic of research, the interplay between the concentration of vanadium ions in the multiphase VO_2_^21–23^, and the role of film thickness, grain size distribution, and crystallinity makes the fabrication highly process-dependent and substrate-dependent^24–26^. Traditionally, deposition techniques used for VO_2_ growth are heavily impacted by oxygen concentration in the growth chamber making repeatability and tunability difficult. Furthermore, large-scale deposition of VO_2_ thin films has been a perennial hurdle for industrial applications. Well-known techniques such as pulsed-laser deposition (PLD) are used to grow crystalline VO_2_; however, PLD cannot cover large surface areas making deposition of thin VO_2_ thin films on large-scale substrates unattainable. PLD technique maintains critical drawbacks of limited deposition uniformity and lack of cluster-free deposition. Although it can be implemented on a variety of substrates such as glass, sapphire and silicon, scaling up is still an obstacle^27^. There are a few reports that study the optimisation of the quality of VO_2_ thin films deposited using the sputtering process^13,28,29^. However, these reports suggest that single crystal substrates such as sapphire are a key requirement for realising high quality VO_2_ thin films, with the desired electrical and optical properties. Other reports utilise sputtering and successfully show VO_2_ thin films on glass and silicon^30,31^. However, substrate-independence and a simultaneous complete characterisation of electrical and optical properties of the same set of thin films is still lacking. As a result, the limitation of substrate choice has restricted applications using VO_2_ despite its unique and versatile characteristics.

This work presents a thin film deposition process by magnetron sputtering to high quality VO_2_ thin films. The process is substrate-independent and highly repeatable. Depositions on three different substrates – glass, quartz, and float-zone silicon (with a resistivity >50 Ω.m) – are carried out to show the flexibility of the process. An IMT where the resistivity reversibly changes by of up to four orders of magnitude (typically three orders of magnitude) is observed for VO_2_ thin films on all three substrates. This is also accompanied by a reversible change in transmission characteristics in the infrared region of the optical spectrum, with >60% discrimination. A systematic analysis of the effects of film thickness and the sputtering gas ratios on the performance of the VO_2_ films is also presented.

## Results

### VO_2_ thin films morphology and composition analysis

**Figure 1:**
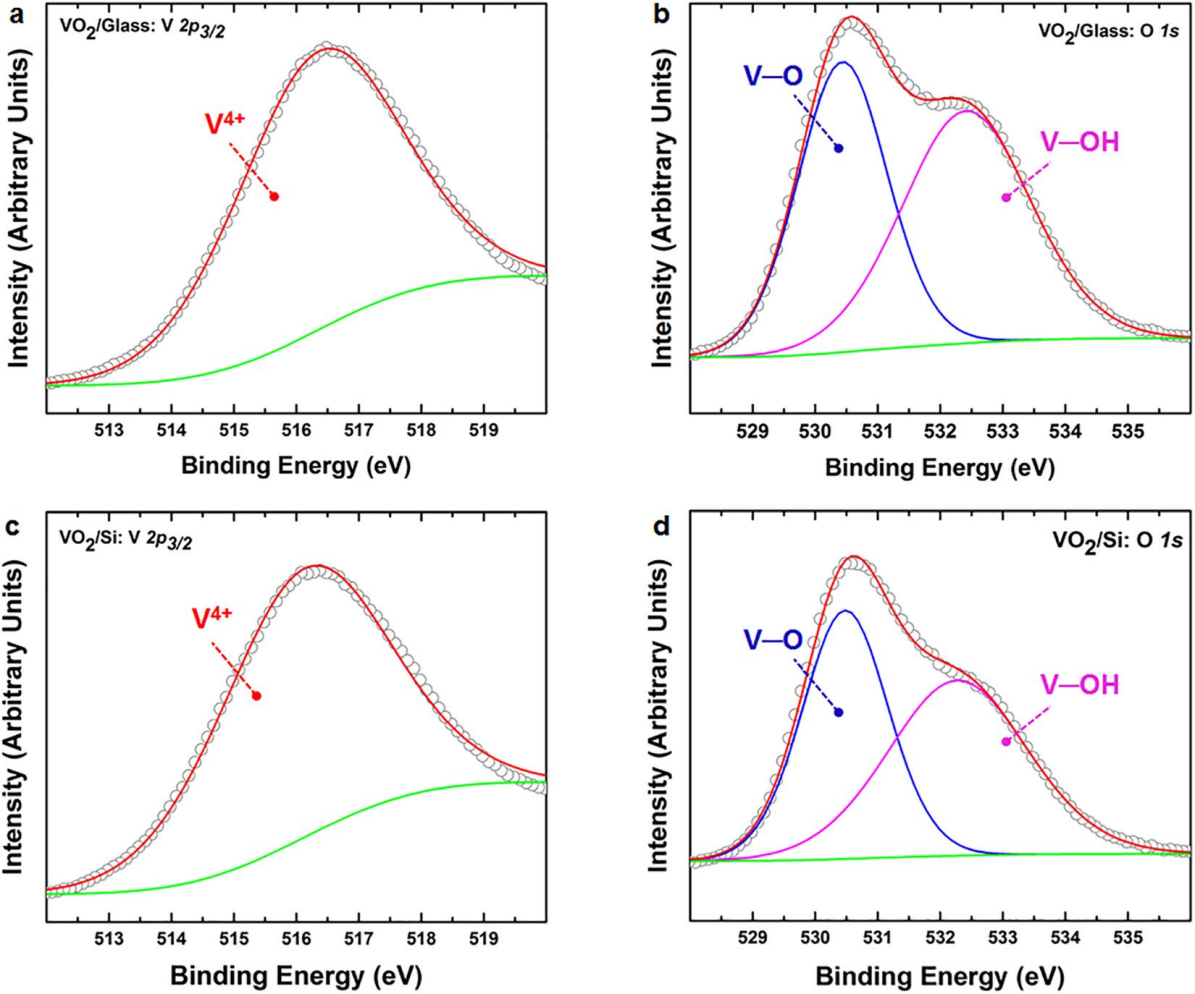
Core-level XPS spectra of vanadium (V 2*p*_3/2_) in (**a**) and (**c**) and oxygen (O 1*s*) in (**b**) and (**d**) collected from post-deposition annealed thin films sputter-deposited on glass and silicon substrates.

To assess the chemical composition of the vanadium oxide thin films and establish presence of the VO_2_ phase, X-ray photoelectron spectroscopic (XPS) analyses are carried out on as-grown and post- annealed thin films. Figure 1 shows high resolution XPS spectra of V 2*p*_3/2_ and O 1*s* collected from the post-deposition annealed thin film. The V 2*p*_3/2_ spectrum in Fig. 1a shows a single peak centered at 516.4 eV which corresponds to the V^4+^ oxidation state^32–34^. The O 1*s* spectrum in Fig. 1b can be fitted with two components with peak positions at 530.4 eV and 532.4 eV attributed to V–O and V–OH, respectively^35^. The combination of the V and O oxidation states confirms the presence of the VO_2_ phase exclusively, given only the V^4+^ oxidation state is observed. Figure 1c and d show similar V2p_3/2_ and O1s peaks for post deposition annealed samples on Si substrates illustrating the substrate independent stoichiometry obtained using our proposed recipe.

The composition of the post-deposition annealed thin film does not show any difference in the oxidation state of vanadium (*i*.*e*., V^4+^) as compared to the as-grown film (See Supplementary information, Figure S1). This indicates that post-deposition annealing only changes the crystallinity of the films without affecting the chemical composition of the sputtered oxide. This was further verified using X-ray diffraction (XRD) studies.

To substantiate a valid comparison between films deposited on different substrates, we do not rely only on composition. Here, we investigate surface morphology and crystallinity using atomic force microscope (AFM) topography scans and X-ray diffraction (XRD) studies.

**Figure 2:**
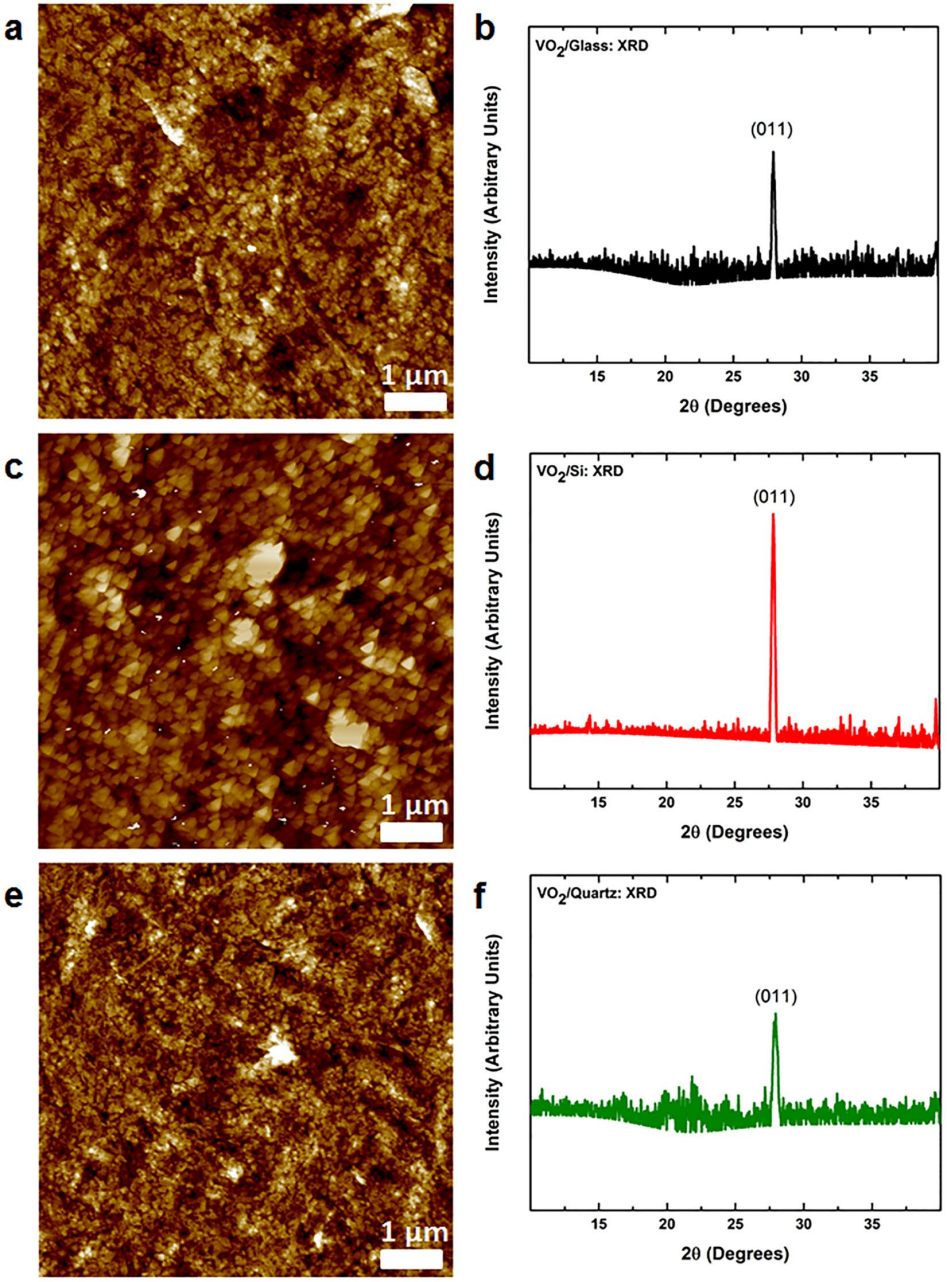
Characterisation of VO_2_ thin films: Atomic force micrographs for post-deposition annealed VO_2_ thin films on (**a**) glass (**c**) high resistivity silicon and (**e**) quartz; X-ray diffractograms for films deposited on (**b**) glass (**d**) high resistivity silicon and (**f**).

Figures 2a,c,e show AFM topography scans of post-deposition annealed VO_2_ thin films for glass, silicon and quartz, respectively. Scans of post-deposition annealed VO_2_ thin films show well-defined grains. The average surface roughness of the films is calculated to be 3.64, 3.39 and 3.86 nm on glass, silicon and quartz respectively. We further investigate our X-ray diffractograms of post-deposition annealed VO_2_ thin films on glass, silicon and quartz substrates. Figure 2b,d and f show X-ray diffractograms for crystalline films on all three substrates. While the room temperature deposited films are amorphous or nanocrystalline with no long-range lattice arrangement observed, the post-deposition annealed films on glass, silicon and quartz show a preferred (011) alignment at a 2θ of ~27.9°, which is characteristic of stoichiometric VO_2_^1,36^. Literature reports indicate that the most effective transition switching in VO_2_ occurs across the (011) and $(\bar{011})$ directions of the monoclinic structure^37^. To gain further insight, full width at half maximum (FWHM) was calculated from the XRD spectra. A FWHM of 0.3, 0.3 and 0.4 for glass, silicon and quartz is obtained. It is known that the FWHM is heavily influenced by discrepancies in crystal structures, however, this is not the case for the films reported in this study as FWHM shows little change for the films deposited on different substrates (See Supplementary information, Figure S2 for FHMH calculations).

Even though as-grown films possess the same composition as post-deposition annealed films they do not display IMT or thermochromism in the infrared region of the optical spectra (See Supplementary information, Figure S3).

**Figure 3:**
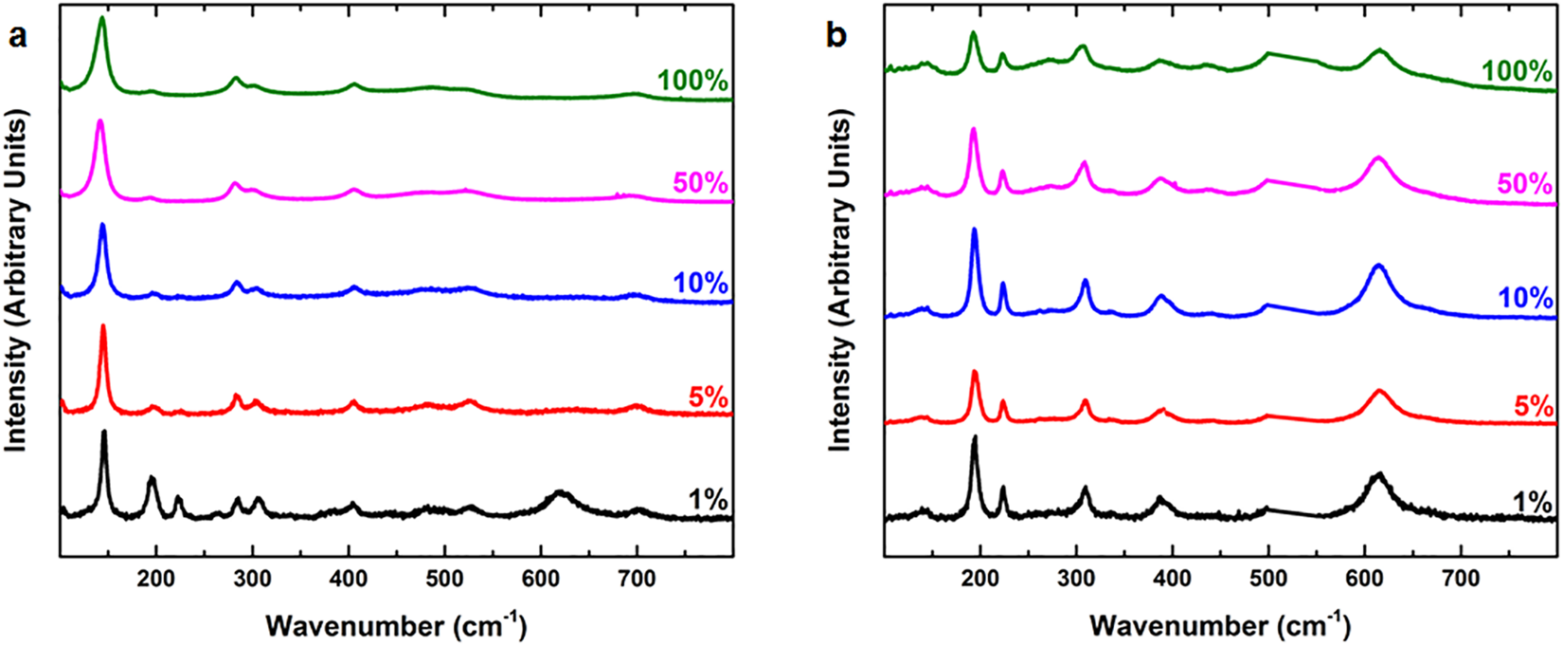
Raman spectrum of the post-deposition annealed VO_2_ thin film fabricated on (**a**) glass and (**b**) silicon acquired with different laser intensities. Percentages correspond to incident power intensities of 532 nm laser, where 100% is 9 mW.

Figure 3 shows the Raman spectra of the post-deposition annealed films fabricated on glass and Si. Peaks and their associated phonon vibrations are summarised in Table 1. These can be assigned to the *A*_g_ and *B*_g_ phonon vibration modes of VO_2_, which is consistent with reports in literature^1,16,38–45^. It should be noted that the 195 and 615 cm^−1^ features that signify the V–O bond vibrations are relatively weak in intensity. We attribute this to the highly localised laser induced heating during the Raman measurements which could induce a phase change of VO_2_ at the measurement location. This phase change is known to significantly reduce the intensity of these vibration modes^36^. Figure 3a,b show the evolution of the Raman spectra with varying incident laser powers. Although Raman results show evidence that the films are indeed VO_2_ since the peaks are consistent with literature (Table 1), the films are affected by localised heating. This effect is discussed in more detail in the discussion section, but since Raman spectroscopy changes the observation of phonon vibrations making absolute conclusion about the full characteristics of VO_2_ thin films on those substrates using only Raman spectroscopy is unreliable. Therefore, we utilise a combination of XPS, AFM and the XRD analysis of the thin films alongside the Raman spectra to form a clearer idea of the nature of VO_2_ films synthesised using our method. AFM topography scans show well-defined grains and an average surface roughness that is consistent on glass, silicon and quartz. XRD crystallinity matching and FWHM indicate similar crystallinity between the VO_2_ films deposited on different substrates reported in this work. The abovementioned compositional, phase and crystallinity characterisations clearly show that the films deposited through the reported recipe are VO_2_ thin films without the presence of any additional phase.

**Table 1:** Raman vibrational modes for the VO_2_ thin films.

Raman Peak	Phonon mode	Reference	Raman Peak	Phonon mode	Reference
140	—	^39, 40, 43– 45^	395	*A*_g_	^39, 40, 43– 45^
195	*A*_g_	^39, 40, 43– 45^	439	*B*_g_	^39, 40, 43– 45^
225	*A*_g_	^39, 40, 43– 45^	489	*B*_g_	^39, 40, 43– 45^
264	*B*_g_	^39, 40, 43– 45^	503	*A*_g_	^39, 40, 43– 45^
280	*—*	^16, 42, 44^	525	*A*_g_	^38, 41^
304	*A*_g_	^39, 40, 43– 45^	585	*B*_g_	^39, 40, 43– 45^
337	*B*_g_	^39, 40, 43– 45^	615	*A*_g_	^39, 40, 43– 45^
385	*A*_g_	^39, 40, 43– 45^	700	*—*	^41, 44^

### Insulator–to–metal transition (IMT), electrical and optical characterization

**Figure 4:**
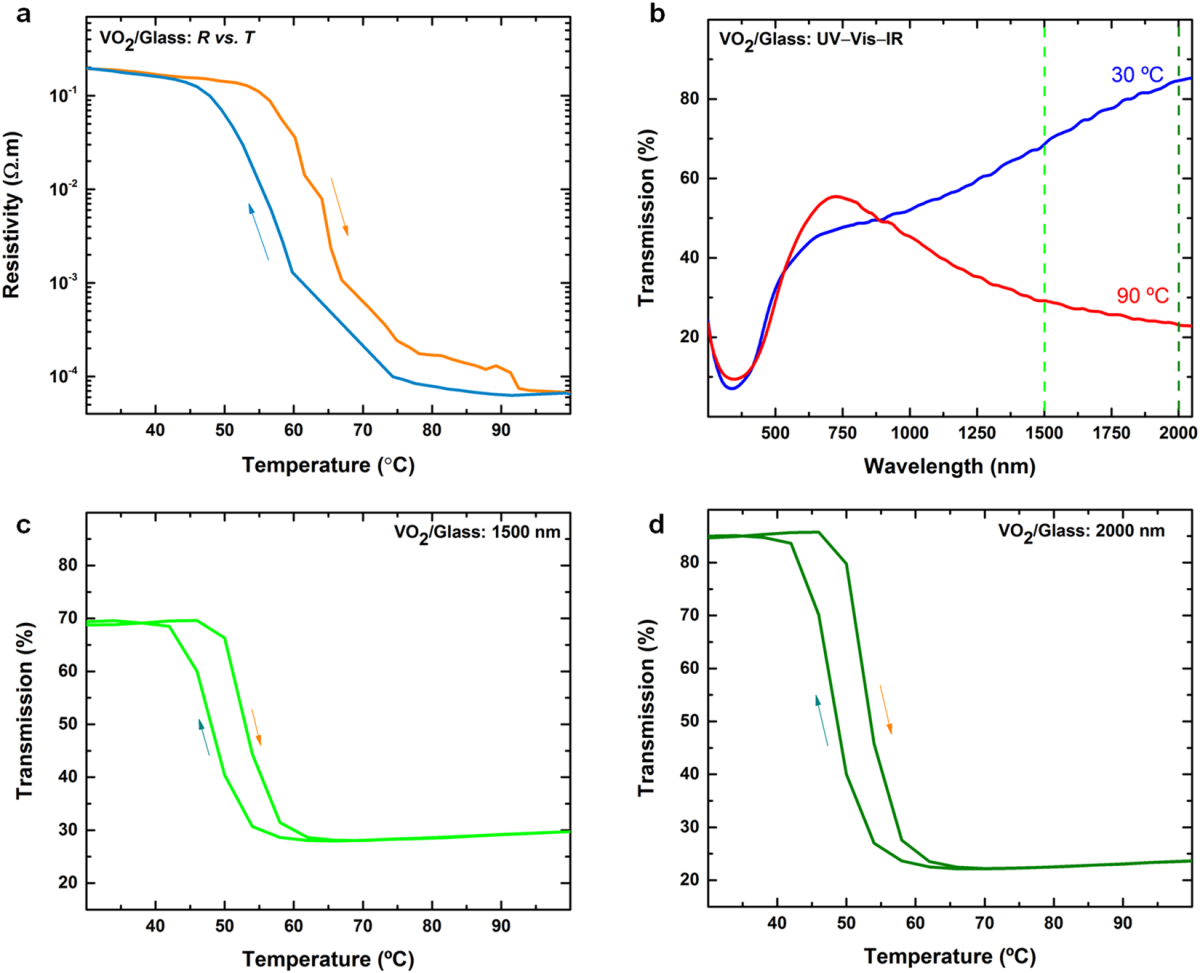
(**a**) Resistivity *vs*. temperature for a VO_2_ thin film on glass. (**b**) Corresponding UV–Vis–IR optical transmission. Optical transmission *vs*. temperature for the VO_2_ thin films on glass acquired at optical wavelengths of (**c**) 1500 nm and (**d**) 2000 nm.

We now characterise the electrical resistivity of the VO_2_ thin films using the four-point probe technique. The temperature-dependent resistivity of the VO_2_ thin films on glass (deposited at 30% Ar:O_2_ ratio for 45 min) is shown in Fig. 4a and shows a drop in resistivity of four orders of magnitude at a temperature of ~69 °C. The amplitude of the IMT is obtained from the resistivity ratio (*R*_90_/*R*_30_) at temperatures of 90 °C and 30 °C, respectively. An obvious thermal hysteresis is also observed between the heating and cooling cycles. Table 2 summarizes the electrical and switching performance for VO_2_ thin films with respect to varying oxygen concentrations and deposition duration. It is seen that the films sputtered at 30% O_2_ for 45 min show the highest magnitude of IMT resistive switching (explaining choice of films for detailed characterisation) indicating a key role played by the oxygen concentration during deposition and the thickness of the film.

**Table 2:** Summary of deposition conditions and the corresponding IMT switching performance.

Oxygen partial pressure (%)	Deposition duration (min)	Film thickness (nm)	Switching ratio *R*_30_/*R*_90_	Rate of change (Ω.m/°C)	Rate of change (Ω.m/min)	Optical switching at 2000 nm (%)
30	15	50.4	1,289	8	32	~40%
30	30	100.8	455	38	150	~58%
30	45	151.2	2,309	28	111	~60%
50	15	31.5	251	15	60	~25%
50	30	63.0	714	7	30	~50%
50	45	94.5	1,433	21	84	~63%

Figure 4b shows a comparison of the optical transmission of a VO_2_ thin film at 30 °C and 90 °C across the ultra-violet, visible, and infrared spectrum (UV–Vis–IR spectroscopy). While the change in the visible range is not significant, the discrimination between the ‘insulator’ (transparent) and ‘metal’ (opaque) states for higher wavelengths is apparent. This agrees with known thermochromic NIR characteristics of VO_2_^4,6,8,9,12,13,18,22,29^. IMT is reversible and repeatable as films display recovery after the removal of the thermal trigger for the IMT. Films undergo consistent and repeatable IMT when heat is re-applied (See Supplementary information, Figure S4).

To further substantiate this observation, temperature-dependent (both heating and cooling cycles) transmission spectra are obtained at two infrared wavelengths of 1500 and 2000 nm and are shown in Fig. 4c,d, respectively. An obvious and significant change in the infrared transmittance can be observed throughout the temperature band where the IMT occurs. From a transmittance of ~70% at 30 °C (insulating phase), the value drops to below 28% and 25% at 90 °C (metallic phase) for 1500 and 2000 nm, respectively. This further elucidates that the optical switching ratios in VO_2_ become larger as we move to longer wavelengths. The optical switching ratios for the various samples fabricated in this study are listed in Table 2. We also note that the transition temperatures for the electrical and the optical measurements are slightly different, which we attribute to discrepancies and collection times between the electrical and optical measurement systems.

According to the Beer–Lambert law, the optical transmittance is exponentially dependent on the film thickness. The data listed in Table 2 confirms that the thickest VO_2_ film exhibits the best electrical and optical switching properties. However, there will be an optimal limit, beyond which the considerable number of charge carriers in the system will inhibit switching owing to scattering at point defects in the crystal lattice. It can be therefore concluded that the infrared switching properties of the VO_2_ thin films depend on thickness as well as the stoichiometry. Additionally, an informed choice of sputtering power needs to be made to obtain a delicate balance between the formation of secondary phases that occurs if the power is too low and poisoning of the high-purity vanadium target if the power is too high. A comparison of electrical and optical properties for the as-grown and annealed VO_2_ films (See Supplementary information, Figure S3) reaffirms that annealing-induced crystallinity and the formation of coalesced grain structure as observed in AFM scans are important for desirable electrical and optical transitions. Post-deposition annealing in our work makes the films crystalline which is important to achieve the characteristic IMT. Such a transition cannot occur on as-deposited amorphous films due to a lack of carriers.

### Substrate-independent VO_2_ thin films

**Figure 5:**
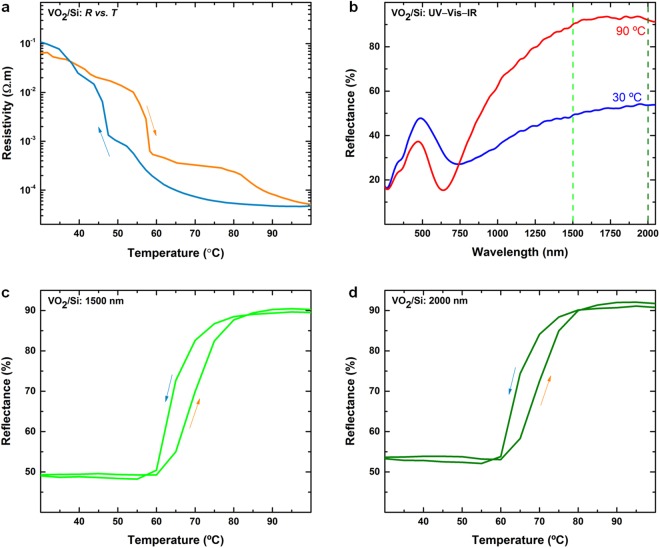
(**a**) Resistivity *vs*. temperature for a VO_2_ thin film on silicon. (**b**) Corresponding UV–Vis–IR reflectance performance. Optical reflectance *vs*. temperature for a VO_2_ thin films on silicon acquired at optical wavelengths of (**c**) 1500 nm and (**d**) 2000 nm.

The ability to synthesise such high-performance films on amorphous glass substrates is a significant outcome. To demonstrate that this synthesis approach is substrate-independent, we perform depositions on silicon and quartz substrates. Electrical and optical characteristics of a VO_2_ thin film deposited on a silicon substrate are shown in Fig. 5. Figure 5a,b shows that the IMT and the optical transition of the thin films are maintained on a silicon substrate. It should be noted that the different electrical properties of silicon affect quality of four-point probe measurements, while reflectivity is measured in place of transmission for UV–Vis–IR spectroscopy. Further variations in the electrical behaviour can also arise from the difference in the thermal properties and the thickness of the substrates. To assess the reflectance at different infrared wavelengths, cyclic reflection data is collected at two infrared wavelengths of 1500 and 2000 nm (as in the case of VO_2_ on glass) and are shown in Fig. 5c,d, respectively. From a reflectance of ~49% (1500 nm) and ~52% (2000 nm) at 30 °C (electrically insulating phase), the value rises to around 90% (1500 nm) and 91% (2000 nm) at 90 °C (metallic phase). It is seen that the electrical IMT switching is at least three orders of magnitude and optical transition is at least 40% irrespective of the substrate.

Similar deposition and characterisation was undertaken with success on quartz substrates to achieve electrical change of 10^4^ and optical discrimination of 55% at 2000 nm (See Supplementary information, Figure S5). Room temperature deposition results in VO_2_ with the correct chemical composition. The major factor affecting the functionality of the metal oxide is the crystallinity and number of oxygen vacancies. It is seen that the transition temperatures for all the substrates lie in a range that is in line with the expectation from the VO_2_ phase^1–6^.

**Figure 6:**
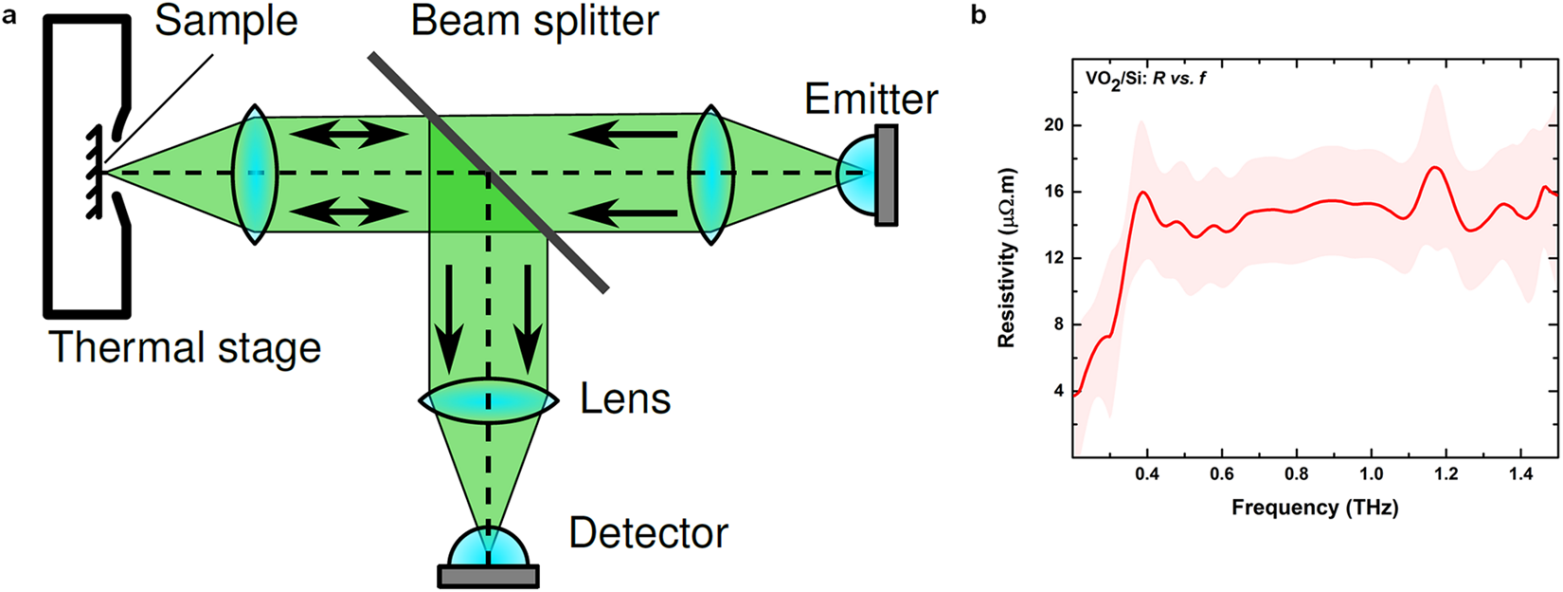
(**a**) Reflective terahertz time-domain spectroscopy setup, where the sample is heated using a thermal stage. (**b**) Extracted resistivity of metallic-phase VO_2_ on Si in the terahertz range, with error bars at one standard deviation shown as a transparent region.

It is well-known that the properties of metals are frequency-dependent^46^. Hence, in order to assess the high-frequency resistivity of the VO_2_ thin films synthesised on this study, we perform characterization at terahertz-range frequencies. Figure 6a shows the terahertz time-domain spectroscopy (THz-TDS) setup used. THz-TDS is an effective technique for extracting carrier concentration and conductive properties of thin films. The VO_2_ thin films on silicon was characterised at high temperature (metallic state). The results are shown in Fig. 6b with resistivity of ~0.5 × 10^−4^ Ω∙m across a frequency range of 0.5–2.5 THz (See Supplementary information, S6 for details of resistivity extraction). This is consistent with the DC resistivity results presented in Figs 4a and 5a. This highlights that VO_2_ retains its key electronic features even at high frequencies, and therefore, is a promising material for applications in the terahertz domain.

## Discussion

We have developed a substrate-independent recipe to deposit crystalline VO_2_ thin films. Using room temperature deposition and low-vacuum post-annealing at 550 °C produces VO_2_ thin films on glass substrates with excellent electrical and optical transition. The careful balance of utilising room temperature deposition to control stoichiometry and post-deposition annealing to define crystalline phase results in single-phase, preferentially-oriented VO_2_ films. The characteristic IMT is verified both electrically and optically with a dramatic change observed in transmission at infrared wavelengths. The versatility of the process is verified by depositing and characterising VO_2_ films on silicon and quartz.

We observe that the thermal conductivity of the substrate plays a crucial role in characterisations measurements. This is evident from the Raman spectra obtained on glass and silicon at different laser powers. The highly localised nature of the laser during the Raman measurements will induce a significant amount of heat which has to be dissipated through the substrate. We conducted a sequential set of measurements with varying Raman laser powers to gain more insight (Fig. 3). For the glass substrate, peaks at 195 and 615 cm^−1^ amongst other peaks disappeared at 100% laser power. This is induced due to the build-up of heat generated due to the laser. On the other hand, the heat dissipates faster on silicon owing to its higher thermal conductivity^47^ compared to glass^48^, which translates to significantly smaller variations in the Raman spectra as shown in Fig. 3. Therefore, Raman spectroscopy cannot be used as a conclusive method of VO_2_ films characterisation especially in the case of substrates with low thermal conductivity. Therefore, we have also used AFM, XRD and XPS to characterise the composition, topography and crystallinity of the VO_2_ thin films which highlight the consistency of the recipe we propose in this study. This also highlights that the certain dissimilarities in both electrical and optical properties in VO_2_ films on Si are not caused due to variations in the quality of the films but rather the thermal properties of the substrate. Alongside thermal properties and substrate thickness it is important to consider that four-point probes also measure the sheet resistance of the underlying substrate which will have an effect in the case of a semiconducting substrate like silicon. This can result in relatively lower IMT magnitudes. It must be noted that the film still maintains up to 3 orders of magnitude without an insulating layer on top of silicon which further supports the fact that the film quality is preserved regardless of the substrate. The previous conclusion is further verified in the case of the quartz substrate, where the characteristics are not different. Since glass and quartz have similar thermal conductivities, the performance of the VO_2_ films fabricated on these substrates are analogous (See Supplementary Information, Figure S5). This substrate-independent method of depositing high quality VO_2_ thin films can open several opportunities for creating smart windows, temperature-tunable memories, modulated nanoplasmonic surfaces, and terahertz devices.

## Methods

### VO_2_ thin film deposition

The VO_2_ thin films are deposited onto plasma-cleaned glass, silicon, and quartz substrates using the pulsed DC magnetron sputtering technique. Detailed parameters used for thin film deposition are shown in Table 3. Subsequently, the as-deposited VO_2_ films are annealed in a furnace, evacuated to low vacuum to achieve a pressure of ~250 mTorr, at 550 °C for 90 min. Several deposition parameters were studied to optimise phase, composition, and switching. These are listed in Table 2, which is discussed in detail. VO_2_ thin films sputtered with 30% O_2_ in an Ar and O_2_ gas mixture for 45 min are ~150 nm thick. All analyses and characterisation discussed in this work pertain to these films.

**Table 3:** Sputtering parameters for depositing VO_2_ thin films using pulsed DC magnetron sputtering.

Target used	Vanadium (99.99%)
Distance from the target	~120 mm
Base pressure	4.0 × 10^−7^ Torr
Sputtering pressure	2.8 × 10^−3^ Torr
Ar:O_2_ flow rate	12.25:5.25 sccm (for 30%)
Sputtering power	200 W
Pulse frequency, reverse time	25 kHz, 5 µs
Substrate temperature	Ambient

### Spectroscopy and microanalysis

X-ray photoelectron spectroscopy (XPS) analysis is conducted using a Thermo Scientific K-Alpha instrument under ultrahigh vacuum (base pressure < 1 × 10^−7^ Pa). An aluminium *K*α X-ray radiation source with energy of 1486.6 eV is used. All core-level spectra of the elements are collected at pass energy of 50 eV and analysed with Advantage software. The binding energies of all principal elements are referenced to the adventitious carbon (C 1s) of binding energy 284.6 eV. Crystallography is verified using a X-ray diffraction powder analyser (D2 Phaser, Bruker). Deposited film thicknesses were measured by stylus profilometry (Dektak XT, Bruker). A Dimension Icon atomic force microscope was used to obtain surface topography. Raman spectra were obtained using a Horiba LabRAM Evolution micro-Raman system equipped with 9 mW, 532 nm laser (0.5 µm lateral resolution, 2 s exposure) and a 50 × objective.

### Insulator–to–metal transition measurements

Electrical measurements were conducted using a Jandel cylindrical four-point probe. UV–Vis–IR transmission and reflection spectra are collected using a CRAIC 20/30 microspectrophotometer. A Linkam stage is used for heating and cooling of the sample during the electrical and optical measurements. The ramp-up and ramp-down rates are controlled at 5 °C/min while measurements are collected.

### Terahertz characterisation

The terahertz-range resistivity of VO_2_ is characterised using a terahertz time-domain spectroscopy (THz-TDS) setup shown in Fig. 6a. A polished high-resistivity silicon wafer is employed as a beam splitter in order to probe the response to normally-incident terahertz radiation^49^, and a Linkam thermal stage is employed to heat the sample and produce the desired phase change. In order to maintain good alignment between the sample and reference measurements, self-referencing is used in this case, where the measurement of the metallic-phase sample is normalised by a corresponding measurement from the insulating-phase counterpart.

## Electronic supplementary material

Additional Supporting Information

## Data Availability

All relevant data is available from the authors on request.
