# Supplementary material for: Insulator–metal transition in substrate-independent VO2 thin film for phase-change devices
Source: Sci Rep. 2017 Dec 20;7:17899. doi: 10.1038/s41598-017-17937-3 (PMC5738395; doi:10.1038/s41598-017-17937-3)
Supplement: Supplementary file 1 — Additional Supporting Information [file 41598_2017_17937_MOESM1_ESM.pdf]

Supporting information for

Insulator–metal transition in substrate-independent VO_2 thin film for phase-change devices

Mohammad Taha*[1], Sumeet Walia[1], Taimur Ahmed[1], Daniel Headland[2], Withawat Withayachumnankul[2], Sharath Sriram[1], and Madhu Bhaskaran*[1]

[1]*Functional Materials and Microsystems Research Group and Micro Nano Research Facility, RMIT University, Melbourne, Victoria 3001, Australia*

E-mail: s3420509@student.rmit.edu.au, madhu.bhaskaran@rmit.edu.au

[2] *School of Electrical and Electronic Engineering, The University of Adelaide, South Australia 5005, Australia.*

S1. As-Deposited VO_2 Thin Films: X-ray Spectroscopy

Figure S1 shows the core-level XPS spectra obtained from the as-grown thin film. The V2$p_{3/2}$ spectrum (Figure S1a) is fitted with one peak centred at 516.8 eV, attribute to V^{4+} oxidation sate. On the other hand, O1s spectrum (Figure S1b) comprises two peaks at 530.7 eV and 531.9 eV which can be associated to the V–O and V–OH bonds. Figure 1c and 1d show similar V2$p_{3/2}$ and O1s peaks for amorphous samples on Si substrates illustrating the substrate independent stoichiometry obtained using our proposed recipe.

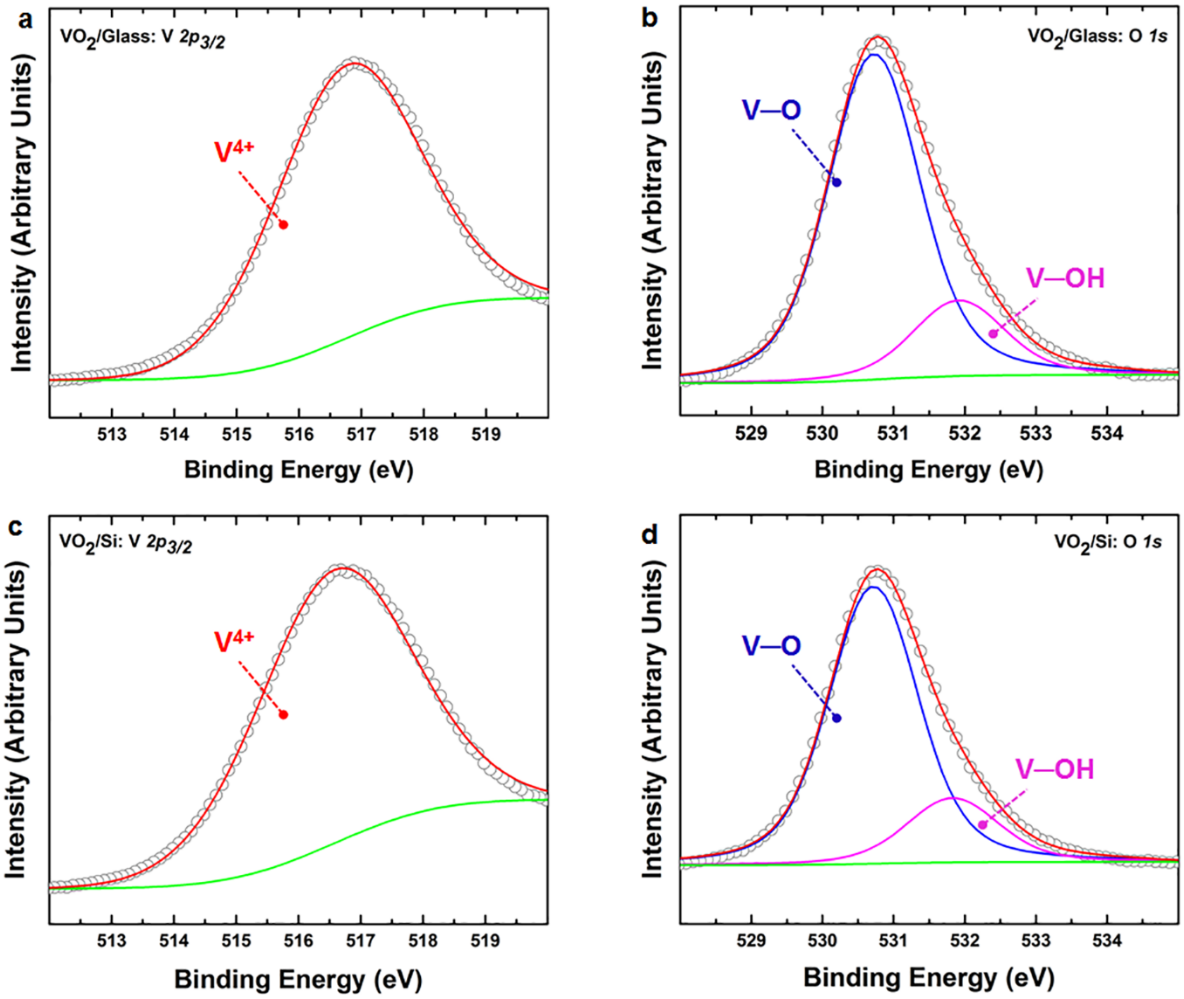

Figure S1. Core-level XPS spectra of V 2$p_{3/2}$ in (a) and (c) and O 1*s* in (b) and (d) collected from as-grown thin films sputtered on glass and Si substrates.

S2. Post-deposition annealed VO_2 Thin Films: X-ray powder diffraction (XRD) and full width at half maximum calculations

Figure S2 shows the high-resolution XRD diffractograms at 2θ of ~27.9º and full width at half maximum (FWHM) for post-deposition annealed VO_2 thin films fabricated on glass, silicon and quartz.

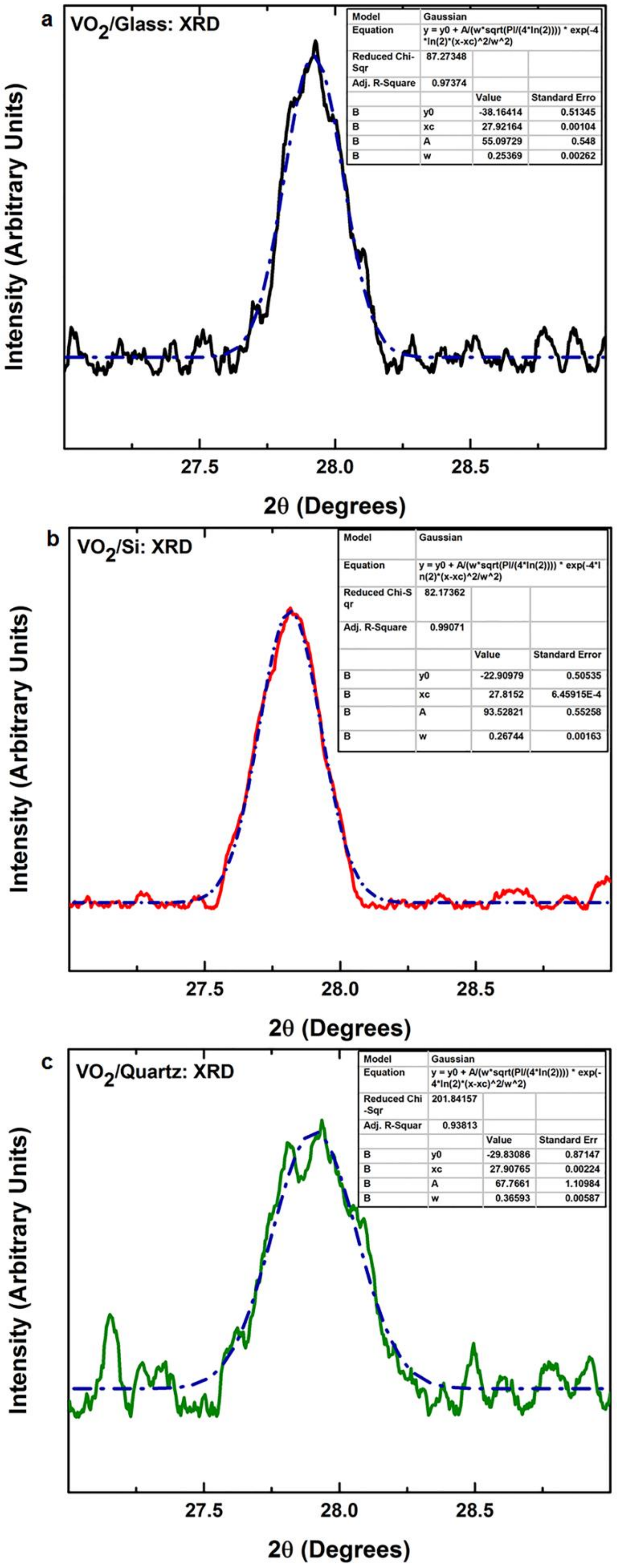
a
VO2/Glass: XRD
Model
Gaussian
Equation
y = y0 + A/(w*sqrt(PI/(4*ln(2)))) * exp(-4*ln(2)*(x-xc)^2/w^2)
Reduced Chi-Sqr
87.27348
Adj. R-Square
0.97374
Value
Standard Erro
B y0 -38.16414 0.51345
B xc 27.92164 0.00104
B A 55.09729 0.548
B w 0.25369 0.00262
Intensity (Arbitrary Units)
27.5
28.0
28.5
2θ (Degrees)
b
VO2/Si: XRD
Model
Gaussian
Equation
y = y0 + A/(w*sqrt(PI/(4*ln(2)))) * exp(-4*ln(2)*(x-xc)^2/w^2)
Reduced Chi-Sqr
82.17362
Adj. R-Square
0.99071
Value
Standard Error
B y0 -22.90979 0.50535
B xc 27.8152 6.45915E-4
B A 93.52821 0.55258
B w 0.26744 0.00163
Intensity (Arbitrary Units)
27.5
28.0
28.5
2θ (Degrees)
c
VO2/Quartz: XRD
Model
Gaussian
Equation
y = y0 + A/(w*sqrt(PI/(4*ln(2)))) * exp(-4*ln(2)*(x-xc)^2/w^2)
Reduced Chi-Sqr
201.84157
Adj. R-Squar
0.93813
Value
Standard Err
B y0 -29.83086 0.87147
B xc 27.90765 0.00224
B A 67.7661 1.10984
B w 0.36593 0.00587
Intensity (Arbitrary Units)
27.5
28.0
28.5
2θ (Degrees)

Figure S2. Full width at half maximum (FWHM) for VO_2 thin films on: (a) glass, (b) silicon and (c) quartz.

S3. As-Deposited VO_2 Thin Films: Electrical and Optical Characterisation

Figure S3 shows the electrical and optical performance of as deposited VO_2 thin films. The films do not show IMT behaviour and their optical performance does not change.

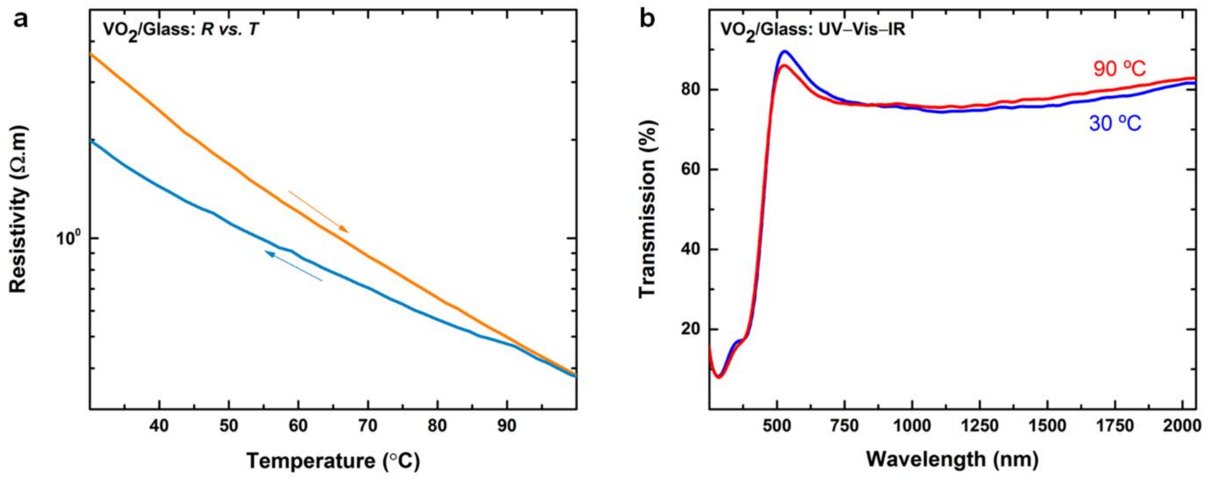

Figure S3. Characterisation of as-grown VO_2 thin films: (a) Resistivity *vs.* temperature and (b) optical transmission.

S4. Repeatability of IMT in VO_2 Thin Films

Figure S4 presents the repeatability and the consistency in the functionality of VO_2 thin films fabricated using this recipe. Figure S4a shows near identical behaviour between Cycle 1 and Cycle 5 of the films being heated and allowed to cool down continuously. This consistency is also apparent in Figure S4b. All cycles in Figure S4b transition in a similar manner with resistivity values showing minimal variation.

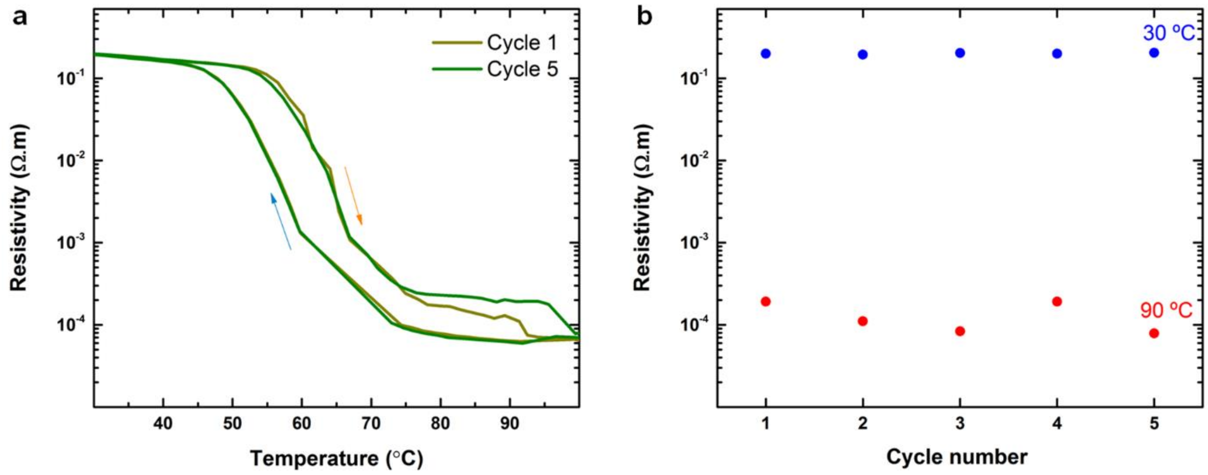

Figure S4. (a) Resistivity *vs.* temperature for the first and fifth sweeps in cyclic testing of post-deposition annealed VO_2 thin films on glass. (b) Consistency in resistivity values for various cycles in the insulator (30 ºC) and metal (90 ºC) states.

S5. IMT in VO_2 Thin Films on Quartz

Figure S5 presents characterisation results for VO_2 thin films deposited on quartz. Figure S5a shows excellent electrical behaviour similar to that on glass and silicon. Figure S5b highlights the optical performance consistent with all the other substrates presented in this work.

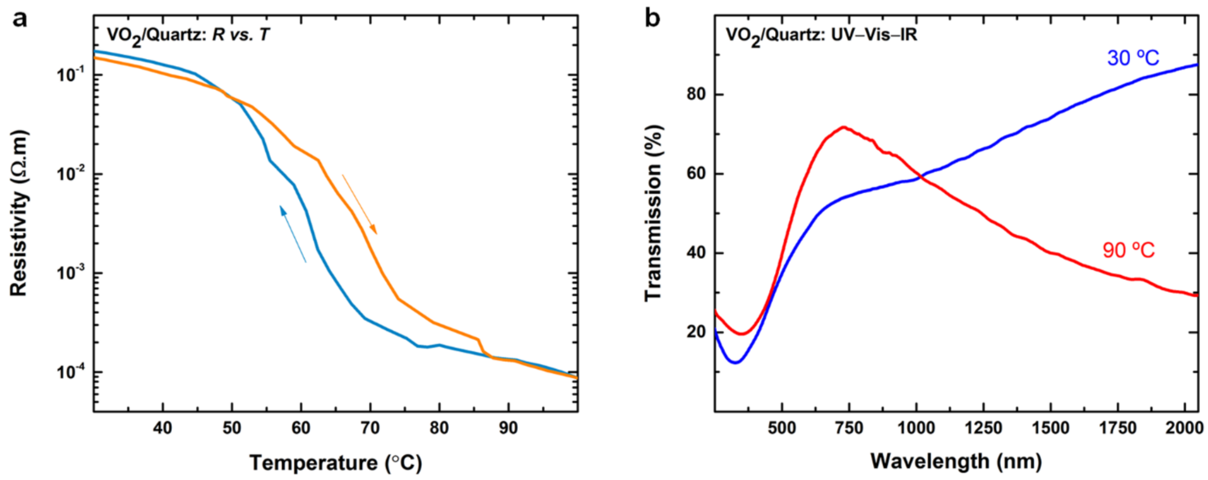

Figure S5. (a) Resistivity *vs.* temperature for a VO_2 thin film on quartz. (b) Corresponding UV–Vis–IR reflectance performance.

S6. Extraction of Terahertz-Range Resistivity

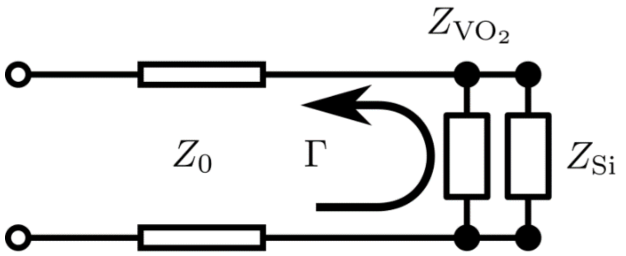

Figure S6. Equivalent circuit model employed to extract the terahertz-range conductivity of VO_2.

A transmission line-based equivalent circuit model is employed to analyse the results of the terahertz time-domain spectroscopy (THz-TDS) measurements. For this model, the air and dielectric media are treated as lengths of transmission line, and the VO_2 thin film is approximated as a lumped shunt impedance. This approximation is valid due to the low electrical length of the film thickness, which is less than 0.2% of a wavelength, and hence any wave propagation effects within the finite-thickness film can be neglected. The value of the shunt impedance is equal to the sheet impedance of the VO_2 layer, $Z_{VO2} = 1/(t\sigma)$, where t is film thickness, and σ is complex conductivity.[1] Time-domain truncation is employed to mitigate confounding factors due to multiple reflection within the Si substrate, and this is possible due to the pulsed-signal nature of THz-TDS systems of this sort.

For the transmission-line circuit model, this is equivalent to perfectly-matching the transmission line that represents the Si substrate, and hence it can be replaced by placing a second shunt impedance, Z_{Si}, in parallel with Z_{VO2}, as opposed to the transmission line length. The value of this shunt impedance is equal to the wave impedance of silicon, $Z_{Si} = Z_0/(\varepsilon_{Si}) \sim 110\ \Omega$. This equivalent circuit model is shown in Figure S, and the reflection coefficient indicated, Γ, represents the reflection of the incident terahertz beam from the VO_2-coated silicon substrate,

$$\Gamma = \frac{Z_{VO_2} \| Z_{Si} - Z_0}{Z_{VO_2} \| Z_{Si} + Z_0}.$$

It is noted that, as VO_2 is a poor conductor in its insulating phase, $Z_{\mathrm{VO2,insulating}} \gg Z_{\mathrm{Si}}$, and hence it will exert negligible impact on the input impedance. As such, the effective impedance of the conducting-phase VO_2 layer can be extracted from the ratio of the measured spectra, $r = E_{\mathrm{metallic}}/E_{\mathrm{insulating}}$, as follows,

$$r = \frac{E_{\mathrm{metallic}}}{E_{\mathrm{insulating}}} = \frac{\Gamma_{\mathrm{metallic}}}{\Gamma_{\mathrm{insulating}}} = \frac{\frac{Z_{VO_2,\mathrm{metallic}} \| Z_{\mathrm{Si}} - Z_0}{Z_{VO_2,\mathrm{metallic}} \| Z_{\mathrm{Si}} - Z_0}}{\frac{Z_{\mathrm{Si}} - Z_0}{Z_{\mathrm{Si}} + Z_0}},$$

$$Z_{\mathrm{VO_2,metallic}} = \frac{-Z_{\mathrm{Si}} Z_0 - {Z_0}^2 - r Z_{\mathrm{Si}} Z_0 + r {Z_0}^2}{r(Z_{\mathrm{Si}} - Z_0) - Z_{\mathrm{Si}} - Z_0}.$$

The terahertz-range resistivity of conducting-phase VO_2 is subsequently extracted from the resulting value of sheet impedance. This procedure is repeated for four VO_2-on-Si samples to construct the error bars, at one standard deviation, that are given in Figure 5b.

References

1 Hansen, R. & Pawlewicz, W. Effective conductivity and microwave reflectivity of thin metallic films. *IEEE Trans. Microwave Theory Tech.* **30**, 2064-2066 (1982).
